# A computational approach for ordering signal transduction pathway components from genomics and proteomics Data

**DOI:** 10.1186/1471-2105-5-158

**Published:** 2004-10-25

**Authors:** Yin Liu, Hongyu Zhao

**Affiliations:** 1 Program of Computational Biology and Bioinformatics, Yale University, New Haven, CT, 06520, USA; 2 Department of Epidemiology and Public Health, Yale University School of Medicine, 60 College Street, New Haven, CT, 06520, USA

## Abstract

**Background:**

Signal transduction is one of the most important biological processes by which cells convert an external signal into a response. Novel computational approaches to mapping proteins onto signaling pathways are needed to fully take advantage of the rapid accumulation of genomic and proteomics information. However, despite their importance, research on signaling pathways reconstruction utilizing large-scale genomics and proteomics information has been limited.

**Results:**

We have developed an approach for predicting the order of signaling pathway components, assuming all the components on the pathways are known. Our method is built on a score function that integrates protein-protein interaction data and microarray gene expression data. Compared to the individual datasets, either protein interactions or gene transcript abundance measurements, the integrated approach leads to better identification of the order of the pathway components.

**Conclusions:**

As demonstrated in our study on the yeast MAPK signaling pathways, the integration analysis of high-throughput genomics and proteomics data can be a powerful means to infer the order of pathway components, enabling the transformation from molecular data into knowledge of cellular mechanisms.

## Background

Signal transduction is the primary means by which eukaryotic cells respond to external signals from their environment and coordinate complex cellular changes. It plays an important role in the control of most fundamental cellular processes including cell proliferation, metabolism, differentiation, and survival [1]. Extracellular signal is transduced into the cell through ligand-receptor binding, followed by the activation of intracellular signaling pathways that involve a series of protein phosphorylation and dephosphorylation, protein-protein interaction, and protein-small molecules interaction.

Recently, with the accumulation of genome sequence information, large-scale genomic and proteomic techniques have offered insights into the components of signal transduction pathways and the molecular and cellular responses to cell signaling. For example, large-scale yeast two-hybrid screening methods and Co-IP technique have been used to identify physical interactions between proteins [2-5]. Synthetic lethal screens are used to identify genetic interactions [6]. The protein chip is an advanced in vitro technique for analyzing protein functions [7]. In addition, microarray experiments can simultaneously measure the transcript abundance of thousands of genes in different conditions. These experimental approaches have generated enormous amounts of data and provide valuable resources for studying signal transduction pathways. However, our understanding of the signal transduction processes underlying these data lags far behind data accumulation. Therefore, there is a great need to develop computational methods to direct biological discovery, enabling biologists to discover the mechanisms underlying complex signaling pathways and interactions among them.

Given the fact that signal transduction is achieved by a cascade of protein interactions and activations, one major challenge in dissecting signal transduction pathways is to determine the order in which the signal is transduced. Traditionally, genetic epistasis analysis is used to address this question. In such analysis, the order of gene function can be determined by comparing the phenotype of a double mutant *ab *to that of a single mutant *a*, or a single mutant *b*. However, this analysis is time-consuming, expensive and sometimes the results can be misinterpreted [8]. Computational methods using large-scale genomics and proteomics information can expand the scope of experimental data and reduce the number of experiments required to detect the order of pathway components. Although it is important, little research has been performed in this field, with a major obstacle being the lack of completeness and accuracy of the data. Here we present a computational approach that integrates different types of information to predict the order of the pathway components assuming all the pathway components are known.

## Results

**Figure 1:**
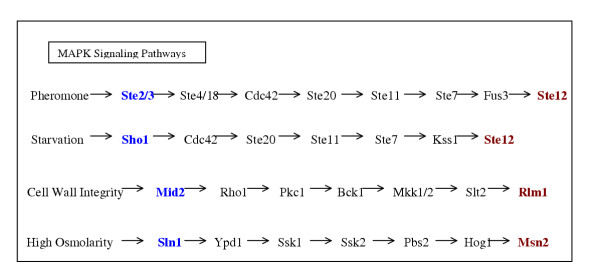
**MAPK signaling pathways in *Saccharomyces cerevisiae*. **Membrane receptors are marked in blue, and transcription factors are marked in red. The figure is adapted from KEGG pathway database [12], and the scaffold proteins and proteins on the pathway branches are omitted for simplicity.

Because the yeast MAPK pathways involved in pheromone response, filamentous growth, maintenance of cell wall integrity and hypertonic shock response are among the most thoroughly studied pathways, we use them to develop and test our method (Fig. 1). As protein-protein interaction plays an important role in achieving the signal transduction process, useful prediction of the order of the pathway components will require knowledge of the interacting partners of these pathway components. Here, we utilize the Database of Interacting Proteins (DIP) that is based on curated collection of all functional linkages of proteins obtained by experimental methods, including yeast two-hybrid experiments, immunoprecipitation, and affinity purification [9]. Although important, the usefulness of the interaction information is limited, as the presence of a physical interaction may not indicate the activation of the interacting proteins. The protein kinases analysis based on protein chip technique provides direct information about protein phosphorylation and activation, but it only presents a very small fraction of the complete picture of protein activation. Compared to the protein chip data, gene expression data from DNA microarray provide an overall picture of whole-cell response under different conditions. Therefore, we utilize this data source as the indirect information about protein activation to complement protein-protein interaction data. Our goal is to develop a computational method for integrating these data sources for ordering yeast MAPK pathway components.

Two expression datasets are used in our analysis, one is composed of 56 conditions relevant to the behavior of MAPK signal transduction and another is the "compendium" set which is composed of 300 diverse mutations and chemical treatments [10,11]. To incorporate the gene expression data, we hypothesize that the genes encoding the proteins on the same signaling pathway, especially the adjacent pathway components, have similar gene expression profiles. In order to test the hypothesis, we calculated the correlations between each pair of genes using the two expression datasets, and performed a hypergeometric test on the similarity of gene expression pattern of the adjacent pathway components. The hypergeometric distribution is given by


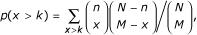


where N represents the total number of protein pairs, M represents the number of protein pairs in adjacent positions on a specific MAPK pathway, n is the total number of protein pairs that have an absolute value of correlation coefficient above a given threshold, e.g. 0.7, and k is the number of adjacent protein pairs having an absolute value of correlation coefficient above this threshold. The p-value obtained from the test is 2 × 10^-4 ^when the threshold is set to 0.7, indicating that protein pairs in adjacent position on a pathway tend to have a higher correlation coefficient value than random protein pairs. This fact is applied in developing our score function that incorporates the gene expression information.

For each MAPK pathway, we examine all permuted orders of the pathway components with the starting point (membrane receptor) and the ending point (transcription factor) of each MPAK pathway fixed and calculate the score for each permutation according to the score function defined as in "Method" section. Then, we rank each permutation based on its corresponding score, with the high-ranking orders being the more likely pathway orders.

**Figure 2:**
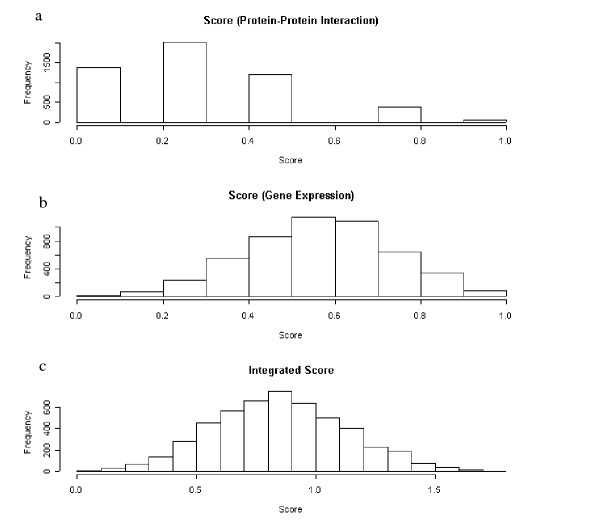
**Distribution of the scores for permuted pheromone response pathways. **(a) Scores based on protein-protein interaction data, (b) Scores based on microarray gene expression data, (c) the integrated scores based on both protein-protein interaction data and gene expression data.

For the pheromone response pathway, the scores based on each individual data set and the scores based on integrating both data sets are shown in Fig. 2. Based on protein-protein interaction data alone, the "true" pathway is assigned a score of 0.75, ranking 241 among all the 5040 possible pathways, while based on gene expression data alone, it is assigned a score of 0.96, ranking 25 among all the 5040 possible pathways. However, after we integrate the scores obtained from two different sources together, the "true" pathway obtain a score of 1.71, with a rank of 2, which is a much higher-ranking than the ranking based on either data type alone. Similar results are shown for the other three yeast MAPK pathways (Table 1). Therefore, our score function that integrates protein-protein interaction data and gene expression data seems to provide more accurate prediction of the order of the pathway components than methods based on either data source alone. This prediction can be used to guide hypothesis-driven research and significantly reduce the number of required experiments.

## Discussion

The rapid accumulation of genomics and proteomics information and the development of large-scale experiment techniques motivate us to develop computational approaches to dissecting different pathways. Arkin *et al*. described a time-lagged correlation analysis to infer the interactions among the components on the first few steps of the glycolytic pathway, thus the order of the components on the glycolytic pathway could be deduced [13]. Schmitt Jr. *et al*. applied this method to identify the cause-effect relationships among genes in the organism *Synechocystis *in response to different light conditions [14]. The limitation of this time-lagged correlation analysis is the requirement of high resolution of time-scales for sampling. That is, if the level of gene expression or the amount of the pathway components is not measured in a small sampling interval, the great resolution into the orderings of pathway components cannot be achieved. Gomez *et al*. used known protein-protein interactions of *Saccharomyces cerevisiae *as training data and represented the proteins as collections of domains to predict links within the human apoptosis pathway [15]. However, not all proteins have a defined domain composition. In principle, these two approaches use either gene expression data or protein-protein interaction data to infer pathways. However, neither method can be applied to jointly analyze data of different sources. Although protein-protein interaction data provide key information to reveal the relationships between components in a singnal transduction pathway, they are subject to many biases (e.g. high false positive and false negative rates) and are not able to capture the dynamic nature of the pathways that are condition dependent. DNA microarray data offer information about whole-cell responses in different conditions but only provide indirect information on the ordering of genes in a specific pathway. These two different data types offer complementary information, and our approach infers the order of the pathway components based on the integration of these two data types and can significantly increase our ability for pathway inference. We note that, despite great improvements over the results based on single data type, our approach is not able to put the correct order as the top one among all possible orders. This is largely due to the imperfectness of current data sources. To further improve our method, we may require data of higher quality or incorporate more types of data, such as protein chip data.

We note that the utility of integrating yeast protein-protein interaction map and gene expression profiles to predict signal transduction network has previously been described by Steffen and colleagues [16]. In their approach, the interaction data were used to create "candidate" pathways and infer the orders between the pathway components, and then the "candidate" pathways were scored according to the number of pathway members that were clustered together based on the expression profiles. However, as many interactions are currently not identified, some links between pathway members may be missing at the very first step and cannot be recovered in the following inference. In addition, the prediction results are highly dependent on the clustering method and the number of clusters into which the genes were grouped. In contrast, our starting point is that we assume that all pathway components are known and use gene expression data to calculate the correlation coefficients between genes and incorporate the results into our score function directly. While our overall objective is somewhat more modest than that of Steffen and colleagues, the motivation of our work was to test whether there is any information in the current data sources to infer the correct order of pathway components. If the goal could not be achieved when all pathway components are known, then it is very unlikely that any method starting from scratch to reconstruct signal transduction pathway will succeed. Fortunately, our results indicate that this modest task can be accomplished and suggest the usefulness of genomics and proteomics information.

We have shown our method can lead to a good prediction for well-known yeast MAPK signaling pathways. In addition, we have tested our approach on the DNA damage checkpoint pathway that is involved in cell-cycle progression. The "true" pathway ranks 4 among all the 750 possible pathways based on our integrated approach, while it has a rank of 46 and 60 based on protein-protein interaction data alone and gene expression data alone, respectively. Therefore, we conjecture that our approach may be applicable to many other pathways including less well-understood ones.

It is worth to note that signaling pathways are not limited to one-dimensional sequence of genes, as our focus in this study. Instead, they should be depicted as multidimensional networks. To make further complicated prediction and modeling of the networks, we need to incorporate more biological information and apply more elaborate statistical approaches.

## Conclusions

We have demonstrated that our integrated approach can significantly improve the performance of predicting the order of signaling pathway components, without detailed knowledge of all the genes in the pathway or the molecular nature of the gene products. It may be important to incorporate other valuable sources of data, including protein chip data, genomic sequence information and protein domain information if we want to make the transition from a linear one dimension pathway to a multidimensional model of signaling networks, which represents a great challenge in the field of systems biology.

## Methods

For protein-protein interaction data, the score function is defined as follows:


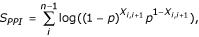


where n is the total number of proteins on the pathway, and X_i, i+1 _= 1 if there is an observed interaction between the i^th ^and the (i+1)^th ^proteins on the pathway and X_i, i+1 _= 0 otherwise. Here p represents the false negative rate of the interaction data. In this study, we fixed the false negative rate as 0.4. It was estimated that the total number of interactions between all yeast proteins or the size of yeast interactome is about 20000~30000 [17,18]. In this study, the interaction data we obtained from DIP includes 15118 pairwise protein-protein interactions, which covers more than 50% of the total number of estimated protein interactions assuming all of the interactions in DIP are true interactions. Indeed, this assumption should be valid as DIP is manually curated and it provides high quality interaction data by minimizing the total number of false positive interactions. Therefore, the false negative rate of the interaction data in DIP may well be less than 0.5. As our method is based on the ranking the of calculated scores, the ranking of all possible orderings are not affected with any false negative rates below 0.5. However, the interaction data availability is limited for some species, for example, only 1379 interactions among about 900 human proteins are included in DIP. In such cases, the performance of our approach may not be as informative as that in yeast.

For gene expression data, the score function is defined as:


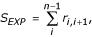


where r_i, i+1 _represents the correlation coefficient between the i^th ^and the (i+1)^th ^proteins on the pathway.

The two data sources are considered with equal importance, so we rescale the score S_i _of all the possible pathways to [0, 1] by


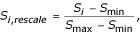


where S_min _and S_max _are the minimum and the maximum scores of all the possible pathways respectively for either protein-protein interaction data or gene expression data. The rescaling procedure is performed on both data sets. The integrated score is the sum of the rescaled scores for each individual data set.

## Authors' contributions

YL designed the study, performed the pathway analysis, and drafted the manuscript. HZ conceived and guided the study. Both authors read and approved the final manuscript.

## Figures and Tables

**Table 1:** A comparison of the prediction results based on using different data types. PPI stands for protein-protein Interaction. The percentile rank of the true pathway is defined as the ratio between the number of pathways having a higher score than the "true" pathway to the number of all permuted pathways.

MAPK pathway	PPI		Expression		PPI + Expression	
	The number of pathways having a higher score	Percentile rank of the true pathway	The number of pathways having a higher score	Percentile rank of the true pathway	The number of pathways having a higher score	Percentile rank of the true pathway

Pheromone Response	240	0.05	24	0.005	1	10^-3.7^
Filamentous Growth	7	0.06	4	0.006	0	0
Cell Wall Integrity	70	0.10	80	0.11	17	0.02
High Osmolarity	0	0	34	0.28	0	0
